# Day-case hip and knee arthroplasty does not increase healthcare system contacts: a prospective multicenter study in a public healthcare setting

**DOI:** 10.2340/17453674.2025.43001

**Published:** 2025-03-18

**Authors:** Abdullahi Abdirisak HIRSI, Oddrún DANIELSEN, Claus VARNUM, Thomas JAKOBSEN, Mikkel Rathsach ANDERSEN, Manuel Josef BIEDER, Søren OVERGAARD, Christoffer Calov JØRGENSEN, Henrik KEHLET, Kirill GROMOV, Martin LINDBERG-LARSEN

**Affiliations:** 1 Center for Fast-track Hip and Knee Replacement, Copenhagen; 2 Department of Orthopaedic Surgery and Traumatology, Odense University Hospital and Svendborg; 3 Department of Orthopaedic Surgery, Lillebaelt Hospital – Vejle; 4 Department of Orthopaedic Surgery, Aalborg University Hospital, Aalborg; 5 Department of Orthopaedic Surgery, Copenhagen University Hospital, Herlev-Gentofte; 6 Department of Orthopaedic Surgery, Næstved, Slagelse and Ringsted Hospitals; 7 Department of Orthopaedic Surgery and Traumatology, Copenhagen University Hospital, Bispebjerg, and Department of Clinical Medicine, Faculty of Health and Medical Sciences, University of Copenhagen; 8 Department of Anaesthesia, Hospital of Northern Zeeland, Hillerød; 9 Section of Surgical Pathophysiology, Copenhagen University Hospital, Rigshospitalet; 10 Department of Orthopaedic Surgery, Hvidovre University Hospital, Hvidovre, Denmark

## Abstract

**Background and purpose:**

Discharge on day of surgery after hip or knee arthroplasty is increasing, but whether this leads to an increase in the overall number of post-discharge healthcare system contacts is unknown. We aimed to investigate whether day-case surgery leads to increased patient-reported healthcare system contacts compared with non-day-case surgery within the first 30 days postoperatively.

**Methods:**

We performed a prospective multicenter study at seven fast-track centers from September 2022 to August 2023. Candidates for primary total hip arthroplasty (THA), total knee arthroplasty (TKA), or unicompartmental knee arthroplasty (UKA) were evaluated for day-case eligibility using pre-defined criteria. Patients received a survey 30 days postoperatively regarding any healthcare system contacts related to surgery. Planned healthcare visits were excluded. We used day-case eligible patients not discharged on day of surgery (inpatients) as control group.

**Results:**

Of 2,278 day-case eligible patients, 2,073 (91%) completed the survey, including 1,146 day-case patients (55%) and 927 inpatients (45%). The overall rate of healthcare system contacts was 49% (95% confidence interval [CI] 45–51) in day-case patients compared with 52% (CI 49–56) in inpatients. Specific contacts included visits to a general practitioner (GP) or out-of-hours medical clinic (25% [CI 22–27] vs 32% [CI 29–35]), the emergency department (ED) (6% [CI 4–7] vs 7% [CI 5–8]), and outpatient clinics or wards (35% [CI 33–38] vs 35% [CI 32–38]). The most common reasons for all types of healthcare contacts were wound problems, prescription renewals, and pain management.

**Conclusion:**

Day-case hip and knee arthroplasties was not associated with increased healthcare system contacts within the first 30 days postoperatively.

There have been significant advancements in the perioperative management of primary hip and knee arthroplasties, particularly with the integration of fast-track protocols [1]. Fast-track surgery is a multidisciplinary approach designed to reduce the surgical stress response, optimize pain control, and enhance postoperative recovery [2]. The ultimate goal of fast-track surgery is to provide day-case surgery safely, where patient recovery is improved enough to enable safe discharge on day of surgery. Furthermore, this may offer societal benefits of reduced hospitalization time and costs, which is much needed in an increasingly strained healthcare system [3-6].

Despite these advancements, an ongoing debate persists regarding the broader impact of such rapid discharge protocols on the overall healthcare system. Previous studies have demonstrated that fast-track protocols, resulting in hospital stays of 1–2 days, do not lead to increased healthcare contacts or readmissions [7,8]. A previous study on day-case surgery found no increase in readmissions within 3 months or additional contact with the discharging department or general practioner (GP); however, this was a retrospective single-center study on 266 patients from the very early phase of the use of day-case surgery (2016–2017) [9,10]. There are no previous prospective multicenter studies from a public healthcare system investigating the impact of day-case protocols on all types of post-discharge healthcare system contacts. Hence, concerns remain that shortened hospital stays could impose a greater burden on other healthcare services, including primary care, emergency department (ED), outpatient clinics, and various hospital departments [11]. Therefore, we aimed to investigate whether day-case surgery leads to increased patient-reported healthcare system contacts within 30 days postoperatively compared with non-day-case surgery. Specifically, we investigated contacts with GP/out-of-hours medical clinic, ED, and orthopedic wards/outpatient clinics.

## Methods

### Study design

This was a prospective multicenter cohort study. The documentation and reporting of the study adhered to the guidelines outlined by the reporting of studies conducted using observational routinely collected data (RECORD) [12].

### Setting

This study was conducted in 7 public arthroplasty centers, performing about 40% of the annual number of hip and knee arthroplasties in Denmark. All centers participated in a multicenter collaboration and adhered to the same protocol for day-case surgery [13]. The duration of the study spanned from September 2022 to August 2023.

### Participants

Patients undergoing primary total hip arthroplasty (THA), total knee arthroplasty (TKA), or unicompartmental knee arthroplasty (UKA) were evaluated for their eligibility for day-case surgery with the inclusion and exclusion criteria detailed in Table 1, see Appendix. Criteria for discharging patients on the same day of surgery are outlined in Table 2, see Appendix. The majority of patients eligible for day-case surgery were included in the day-case pathway and went home on the day of surgery. However, for various reasons [14] a number of day-case eligible patients were not discharged on the day of surgery, forming the control group (inpatients). The choice of control group was based on the premise that the 2 groups were comparable in baseline characteristics including age, clinical frailty scale, body mass index (BMI), civil status, and medical comorbidities due to the initial screening for day-case eligibility (Table 1, see Appendix).

**Table 1:** Inclusion and exclusion criteria for planned discharge on day of surgery

**Inclusion criteria**
Unilateral elective primary THA, TKA, or UKA Age 18–80
**Exclusion criteria**
Acute myocardial infarction, cerebrovascular accident, transient ischemic attack, or coronary atherosclerotic disease within last 3 months Unstable ischemic heart disease Ejection fraction < 40% Glomerular filtration rate < 60 mL/min/1.73 m2 Chronic obstructive pulmonary disease with home oxygen Insulin-dependent diabetes mellitus Sleep apnea requiring mechanical treatment CFS ≥ 4 **^a^** 2 or more falls within last 3 months Body mass index < 18.5 or > 40 Not interested in discharge on day of surgery No adult present at home during the initial postoperative night **^b^**

a CFS = Clinical Frailty Scale [21].

b This criterion was inadvertently omitted from the protocol paper but has consistently been applied across all centers.

**Table 2:** Criteria for discharge on day of surgery

Activity level
Steady gait with crutches No dizziness during mobilization Can use stairs, if required by participant’s home environment
Postoperative nausea and vomiting (PONV)
Minimal and efficiently treated with or without medication EWS **^a^** < 2 Patients with EWS ≥ 2 must be discussed with a doctor prior to discharge
Pain
Numeral rating scale (NRS) (0–10, with 0 being no pain and 10 being the worst pain imaginable) NRS < 3 at rest NRS < 5 when walking 5 meters Or otherwise, acceptable level of pain assessed by the participant regardless of NRS score
Postoperative bleeding
Should be consistent with expected blood loss for this procedure and not require repeated dressing change

a EWS = national implemented Early Warning Score systems based on NEWS2 from the Royal College of Physicians [22].

### Data sources

Dedicated research teams at each center conducted prospective data collection, with the possibility of physician assistance if needed. Subsequently the data was stored in an online REDCap database (https://project-redcap.org/), facilitated through a collaboration with the Open Patient data Explorative Network (OPEN) at Odense University Hospital [15]. Outcome data was available from an electronic questionnaire sent out 30 days postoperatively inquiring if they had contacted their GP/out-of-hours medical clinic, ED, or orthopedic ward/outpatient clinic. The out-of-hours medical clinic is a publicly available service in Denmark where patients are directly connected to a healthcare professional, usually a general practitioner. The healthcare professional then determines whether the issue can be addressed via telephone consultation or if further examination is necessary at an out-of-hours medical helpline clinic or an emergency department. The questionnaire also requested details on the reasons for these contacts, if any.

### Outcome

The primary outcome was the overall rate of patient-reported contacts with the healthcare system within 30 days postoperatively. Secondary outcomes included specific patient-reported contacts with GP/out-of-hours medical clinic, ED, or orthopedic ward/outpatient clinics. Contacts related to scheduled appointments or issues not associated with the surgery were excluded from the analysis. Responses regarding the reasons for healthcare contacts were initially categorized by the first author, AAH, and then reviewed by the senior author, MLL. For the rate of contact with the healthcare system, we measured the proportion of individuals in each group who had contact with the healthcare system on at least 1 occasion, irrespective of the total number of contacts. For specific reasons for contact (Figures 2–4), we documented the reason for each interaction with the healthcare system, allowing for the possibility that a single individual with multiple visits could have more than 1 reason.

### Statistics

Continuous variables are specified as the mean with standard deviation (SD) and range, while categorical variables are described using absolute and relative frequencies. Outcomes are presented as proportions with 95% confidence intervals (CI). RStudio 2023.09.1 software (R Foundation for Statistical Computing, Vienna, Austria) was utilized for data analysis.

### Ethics, funding, use of AI, and disclosures

Funding for “The Center for Fast-track Hip and Knee Replacement” collaboration was secured in 2021 from the NOVO Nordisk Foundation (Grant number: NNF21SA0073760). This funding included provisions for research staff across all participating centers, data management, and monitoring of complications. The overall fast-track project was preregistered at ClinicalTrials.gov (NCT05613439) and within the Region of Southern Denmark, receiving the necessary data processing approval (Journal No 22/39454).

AI tools were not used in preparation of this manuscript.

OD received partial salary funding from the Candys Foundation. CV received travel expenses from Stryker paid to an institution with no relevance to the present study.

Given that the treatment of patients eligible for outpatient surgery adhered to the standard of care at these centers, as outlined in the specified guideline [13], ethical approval was unnecessary according to Danish law. All authors are integral members of the steering committee for the Centre for Fast-track Hip and Knee Replacement and have declared no conflicts of interest pertaining to this study. Complete disclosure of interest forms according to ICMJE are available on the article page, doi: 10.2340/17453674.2025.43001

## Results

**Figure 1:**
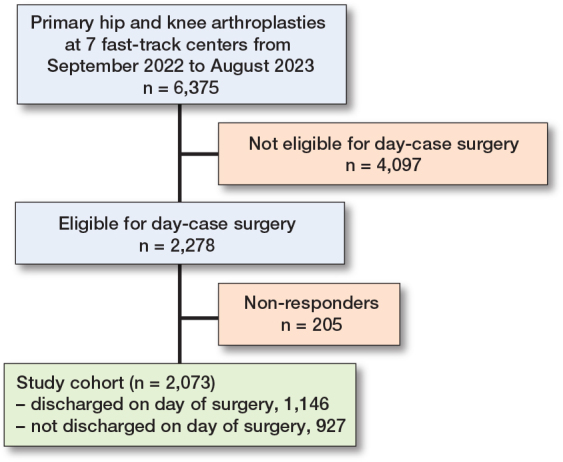
Flowchart showing patient inclusion in the study.

During the study period, 6,375 patients underwent hip or knee arthroplasty. Among them, 2,278 patients were eligible for day-case surgery and 91% completed the survey and were analyzed; 1,146 (55%) were discharged on the day of surgery, while 927 (45%) were not (inpatients). 40% patients underwent THA, 36% TKA, and 25% UKA (Figure 1).

The patient demographics were similar between groups, except for a higher proportion of females in the inpatient group (56% vs 48%) (Table 3).

**Table 3:** Patient demographics. Values are count (%) unless otherwise specified

Factor	Entire study cohort (n = 2,073)	Patients discharged on the day of surgery (n = 1,146)	Patients not discharged on the day of surgery (n = 927)
Surgical procedure			
THA	829 (40)	432 (38)	397 (43)
TKA	738 (36)	388 (34)	350 (38)
UKA	506 (24)	326 (28)	180 (19)
Mean age (SD) [range]	66 (9) [27–80]	66 (9) [32–80]	66 (9) [27–80]
Sex			
Females	1,068 (52)	550 (48)	518 (56)
Males	1,005 (48)	596 (52)	409 (44)
Mean BMI (SD) [range]	28 (4) [19–9]	28 (4) [19–39]	29 (5) [19–39]
Cohabitation			
Cohabiting	1,832 (89)	1,021 (90)	811 (88)
Living alone	228 (11)	118 (10)	110 (12)
Unknown	13 (0.6)	7 (0.6)	6 (0.6)
Mean CFS (SD) [range]	2 (0.7) [1–3]	2 (0.7) [1–3]	2 (0.7) [1–3]
Pharmacologically treated			
diabetes mellitus	117 (5.6)	62 (5.4)	55 (5.9)
heart disease	433 (21)	216 (19)	217 (23)
pulmonary disease	88 (4.2)	47 (4.1)	41 (4.4)

BMI = Body Mass Index; CFS = Clinical Frailty Scale [21].

### All healthcare contacts

Analysis of all healthcare contacts within 30 days post-surgery showed that 49% (CI 45–51) of day-case patients had one or more contacts with the healthcare system, compared with 52% (CI 49–56) in the inpatient group.

### Contacts with GP or out-of-hours medical clinic

**Figure 2:**
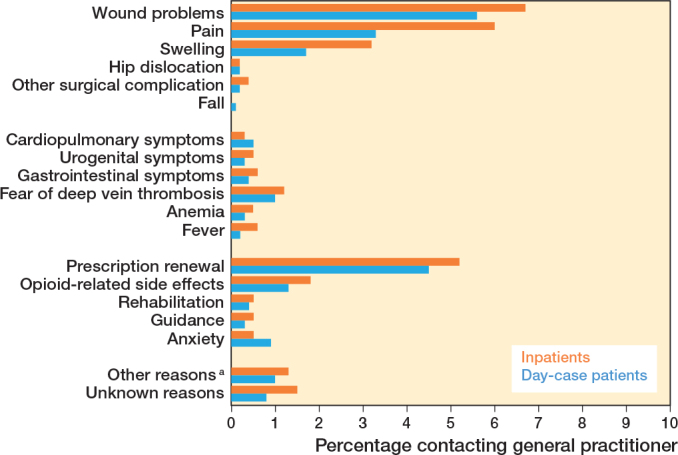
Reasons for contacting general practitioner or out-of-hours medical clinic call (%). Percentages represent each contact reason as a proportion of the total number of patients. ^a^ Other reasons = blood tests, sleep disturbances, back pain, etc.

25% (CI 22–27) of day-case patients contacted their GP or out-of-hours medical clinic, vs 32% (CI 29–35) in the inpatient group. The primary reasons for GP or out-of-hours medical clinic included wound problems (6% day-case patients [CI 4–7] vs 7% inpatients [CI 5–8] ), prescription renewals (5% day-case patients [CI 3–6] vs 5% inpatients [CI 4–7]), and pain management (3% day-case [CI 2–4] vs 6% inpatients [CI 5–8]) (Figure 2).

### Contacts with emergency department

**Figure 3:**
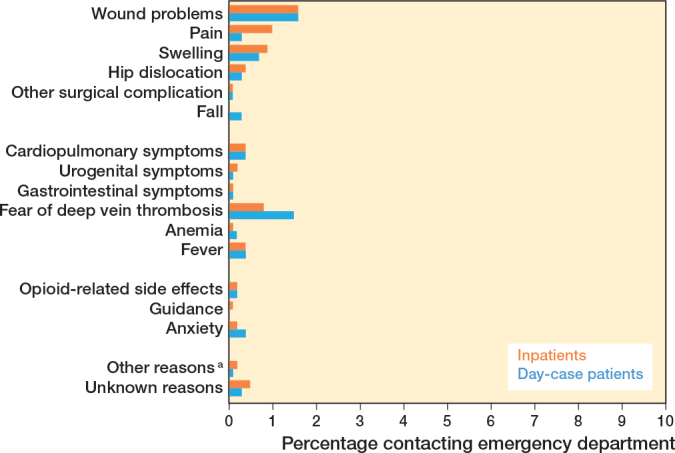
Reasons for contacting the emergency department (%). Percentages represent each contact reason as a proportion of the total number of patients. ^a^ Other reasons = electrolyte imbalance, penicillin side effects, etc.

Emergency department contacts were similar between the groups, with 6% (CI 4–7) in the day-case group vs 7% (CI 5–8) in the inpatient group. Emergency department visits were primarily for wound problems (2% day-case [CI 0.9–2] vs 2% inpatients [CI 0.8–2]), deep vein thrombosis (DVT) suspicion (2% day-case [CI 0.8–2] vs 0.7% inpatients [CI 0.2–1]) and swelling (0.8% day-case [CI 0.2–1] vs 0.8% inpatients [CI 0.3–1]) (Figure 3).

### Contacts with orthopedic wards and outpatient clinics

**Figure 4:**
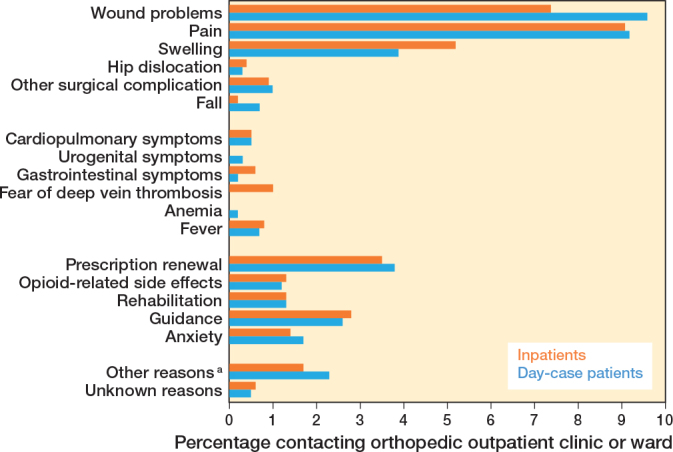
Reasons for contacting the orthopedic outpatient clinic or ward (%). Percentages represent each contact reason as a proportion of the total number of patients. a Other reasons = sleep disturbances, allergy skin staplers, etc.

Contacts with orthopedic wards or outpatient clinics were also comparable, with 35% (CI 33–38) of day-case patients vs 35% (CI 32–38) of inpatients seeking contact. Outpatient clinic or ward contacts were mostly due to pain (9% day-case [CI 8–11] vs 9% inpatients [CI 7–11]), wound problems (10% day-case [CI 8–11] vs 7% inpatients [CI 6–9]) and swelling (4% day-case [CI 3–5] vs 5% inpatients [CI 4–7]) (Figure 4).

### Healthcare contacts by arthroplasty procedure type

Patients undergoing TKA had the highest overall rate of healthcare contacts, with 57% (CI 52–62) in day-case patients and 60% (CI 55–65) in inpatients. This was followed by UKA, where both day-case patients and inpatients reported similar contact rates of 49% (CI 44–55) and 49% (CI 42–57), respectively. THA patients had the lowest rates of healthcare contacts, with 38% (CI 34–43) in day-case patients and 48% (CI 43–53) in inpatients (Table 4).

**Table 4:** Healthcare contacts by arthroplasty procedure type. Values are count (%) and [95% confidence interval]

Factor	THA n = 829		TKA n = 738		UKA n = 506	
	Day-case patients 432 (52)	Inpatients 397 (48)	Day-case patients 388 (53)	Inpatients 350 (47)	Day-case patients 326 (64)	Inpatients 180 (36)
All healthcare contacts	166 (38) [34–43]	189 (48) [43–53]	233 (57) [52–62]	210 (60) [55–65]	160 (49) [44–55]	89 (49) [42–57]
Contacts with general practioner or out-of-hours medical clinic	90 (21) [17–25]	115 (29) [25–34]	122 (31) [27–36]	140 (40) [35–45]	73 (22) [18–27]	42 (23) [18–30]
Contacts with emergency department	22 (5) [3–8]	19 (5) [3–8]	24 (6) [4–9]	28 (8) [5–11]	19 (6) [4–9]	13 (7) [4–12]
Contacts with orthopedic wards and outpatient clinics	115 (27) [23–31]	129 (32) [28–37]	174 (45) [40–50]	132 (38) [33–43]	116 (36) [30–41]	64 (36) [29–43]

## Discussion

We aimed to investigate whether day-case surgery leads to increased patient-reported healthcare system contacts compared with non-day-case surgery within the first 30 days postoperatively. We found that day-case hip or knee arthroplasty did not lead to increased healthcare contacts compared with inpatients with similar preoperative characteristics. This finding is encouraging and refutes concerns that we are shifting contacts to other parts of the healthcare system by discharging patients on the day of surgery.

Our data reveals that a high number of patients contact their GP or out-of-hours medical clinic regardless of whether they were discharged on the same day or stayed overnight. This likely reflects the GP’s role as the primary healthcare contact in Denmark. To our knowledge only one previous study has explored contacts with general practitioners and doctors on call after day-case arthroplasty surgery, supporting our results despite a smaller sample size (n = 261) and the single-center setting of their study [10].

Wound complications were the primary reason for healthcare contacts in both day-cases and inpatients, ranging from bleeding to signs of infection. Prescription renewals, especially for analgesics, were frequent, encompassing scenarios where the clinic had not issued the prescription for medication initially or where patients had finished their prescribed supply. Although prescription management is not a patient morbidity issue, it does consume significant physician time. This finding also reveals room for improvement in postoperative pain management as the initial pain medication plan may not have been sufficient on discharge regardless of whether the patients were discharged on day of surgery or not. Interestingly, the inpatients had more contacts with the GP or out-of-hours medical clinic for pain management (3% day-case [CI 2–4] vs 6% inpatients [CI 5–8]). The cause of this disparity is unclear, but it may indicate a confounding factor that necessitated overnight stays for some of the otherwise day-case eligible patients.

ED contacts were low across both groups in our study, with wound issues being the predominant reason. Additionally, concerns about swelling prompted some patients to contact the emergency department. A significant number of emergency department visits were motivated by fear of DVT, despite the very low risk (0.4%) of this condition as reported in several prior publications [16,17]. Treu et al. [18], in a retrospective single-center study, reported similar findings. They also identified pain and swelling, wound complications, and concerns about DVT as the most frequent reasons for emergency department visits, reflecting the trends observed in our study.

Contacts with orthopedic wards or outpatient clinics were comparable between the groups in our study. Pain was the predominant reason for seeking care, followed by wound complications and swelling. There is limited research investigating the frequency of contacts with orthopedic departments and outpatient clinics. In a 4-week follow-up study, Goyal et al. [4] found no significant differences in the average number of contacts between day-case and inpatient groups. This observation is consistent with our results.

### Strengths

To our knowledge, the current study is the first study investigating the patient-reported causes of different healthcare contacts following outpatient hip or knee arthroplasty in a multicenter setting from a public healthcare system. The current study’s strengths primarily lie in its prospective design and standardized multicenter setup, which adheres to a common, well-defined day-case protocol. Furthermore, the cohort reported on is the largest by far on this subject. We also achieved a high response rate (91%), and both responders and non-responders were similar in baseline characteristics, which enhances the generalizability and reliability of the findings (Table 5, see Appendix).

### Limitations

As the results are based on patient-reported data, this may introduce recall bias, potentially affecting the accuracy of healthcare contact reports. Another limitation is that we acknowledge that our control group is not ideal because it consisted of patients found eligible for day-case surgery, but for various reasons they were not discharged on day of surgery. However, in another study on the same patient cohort, we found that logistical reasons, such as eligible patients being scheduled late for surgery with resulting late return to ward rather than medical or clinical reasons, were one of the main reasons for no same-day discharge [14]. Therefore, we found this group to be the most appropriate control group. The alternative was to select a control group of patients not eligible for day-case surgery, but that would involve individuals with more severe health issues, making them less relevant for this study’s objectives.

Another limitation is that we did not account for all other healthcare contacts, such as additional home care and other municipal services. However, we find this less relevant, because our study group is generally healthy and unlikely to use these additional services. As this study was conducted within the Danish public healthcare setting, the findings may have limited applicability to countries with differing healthcare structures.

Contacts were generally high in both groups, putting a strain on the healthcare system. Taking into account that this cohort was generally healthy, broadening the criteria to less healthy patients would likely result in higher healthcare contacts. TKA was associated with the highest rate of postoperative healthcare contacts. This finding likely reflects the unique challenges posed by knee surgery, including higher incidences of wound complications, persistent pain, and swelling, which are more pronounced compared with hip procedures [19].

Future studies should focus on strategies to reduce these contacts. One promising approach is incorporating telecommunication. For example, a recent study showed a 29% reduction in contacts through a team-based digital communication intervention (eDialogue) [20]. Additionally, enhancing patient education before discharge could mitigate unnecessary contacts by providing clearer information on postoperative care and when to seek medical help. These measures could help streamline postoperative care and alleviate the burden on healthcare resources.

### Conclusion

We found that day-case hip and knee arthroplasties were not associated with increased healthcare system contacts. These findings contribute to the growing body of evidence supporting the efficacy and safety of day-case surgery in orthopedics, encouraging further adoption of such protocols.

## Notes

CV, TJ, MRA, MJB, SO, CCJ, HK, KG, and MLL are members of the Centre for Fast-track Hip and Knee Replacement steering committee. The synopsis of this study was planned and accepted at steering committee meetings. AAH and OD undertook data gathering and processing. AAH, OD, and MLL drafted the manuscript. All authors critically reviewed the manuscript and accepted the final version.  

Handling co-editor: Bart A Swierstra

*Acta* thanks Justine M Naylor and Martijn Schotanus for help with peer review of this manuscript.

## Appendix

**Supplementary Table 3:** Baseline characteristics of responders and non-responders. Values are count (%) unless otherwise specified

Factor	Responders (n= 2,073)	Non-responders (n = 205)
Day-case patients	1,146 (55)	115 (56)
Surgical procedure		
THA	829 (40)	94 (46)
TKA	738 (36)	48 (25)
UKA	506 (24)	63 (29)
Mean age (SD) [range]	66 (9) [27–80]	65 (10) [31–80]
Sex		
Females	1,068 (52)	107 (52)
Males	1,005 (48)	98 (48)
Mean BMI (SD) [range]	28 (4) [19–39]	28 (4) [19–39]
Cohabitation		
Cohabiting	1,832 (89)	178 (87)
Living alone	228 (11)	26 (13)
Unknown	13 (0.6)	1 (0.5)
Mean CFS (SD) [range]	2 (0.7) [1–3]	2 (0.7) [1–3]

BMI = body mass index.

CFS = Clinical Frailty Scale [21].
